# Microbiome in Cancer Development and Treatment

**DOI:** 10.3390/microorganisms12010024

**Published:** 2023-12-22

**Authors:** Sona Ciernikova, Aneta Sevcikova, Beata Mladosievicova, Michal Mego

**Affiliations:** 1 Department of Genetics, Cancer Research Institute, Biomedical Research Center of the Slovak Academy of Sciences, Dubravska cesta 9, 845 05 Bratislava, Slovakia; aneta.sevcikova@savba.sk; 2 Institute of Pathological Physiology, Faculty of Medicine, Comenius University, Sasinkova 4, 811 08 Bratislava, Slovakia; beata.mladosievicova@fmed.uniba.sk; 3 2nd Department of Oncology, Faculty of Medicine, Comenius University and National Cancer Institute, 833 10 Bratislava, Slovakia; misomego@gmail.com

**Keywords:** the gut microbiome, dysbiosis, cancer treatment efficacy, late effects, cognitive impairment, cardiotoxicity, probiotics, fecal microbiota transplantation

## Abstract

Targeting the microbiome, microbiota-derived metabolites, and related pathways represents a significant challenge in oncology. Microbiome analyses have confirmed the negative impact of cancer treatment on gut homeostasis, resulting in acute dysbiosis and severe complications, including massive inflammatory immune response, mucosal barrier disruption, and bacterial translocation across the gut epithelium. Moreover, recent studies revealed the relationship between an imbalance in the gut microbiome and treatment-related toxicity. In this review, we provide current insights into the role of the microbiome in tumor development and the impact of gut and tumor microbiomes on chemo- and immunotherapy efficacy, as well as treatment-induced late effects, including cognitive impairment and cardiotoxicity. As discussed, microbiota modulation via probiotic supplementation and fecal microbiota transplantation represents a new trend in cancer patient care, aiming to increase bacterial diversity, alleviate acute and long-term treatment-induced toxicity, and improve the response to various treatment modalities. However, a more detailed understanding of the complex relationship between the microbiome and host can significantly contribute to integrating a microbiome-based approach into clinical practice. Determination of causal correlations might lead to the identification of clinically relevant diagnostic and prognostic microbial biomarkers. Notably, restoration of intestinal homeostasis could contribute to optimizing treatment efficacy and improving cancer patient outcomes.

## 1. Introduction

Exploring the role of the microbiome in cancer has become an important research area, leading to the discovery of mechanisms by which particular bacteria influence the etiopathogenesis of various malignancies. Currently, preclinical and clinical studies are accumulating, confirming the significant impact of the gut and tumor microbiome on the efficacy and occurrence of adverse effects of anti-tumor therapy. Microorganisms inhabiting tumors constitute a crucial part of the tumor microenvironment, influencing tumor development and progression [1]. Tumor microbiome, malignant cells, and non-malignant compartments represent integral parts of the tumor microenvironment [2]. Reactive oxygen species (ROS), produced by cancer cells, myeloid-derived suppressor cells, regulatory T cells (Treg), and tumor-associated macrophages, reduce immune responses [3]. Moreover, specific bacterial members within tumors trigger ROS production by cells within the tumor microenvironment [4].

Specific diet components can also induce ROS production. As Liu et al. observed, n-3 fatty acids in fish oil have induced ROS production and macrophage death. A supplementation with cocoa butter high-fat diet (HFD) induced obesity and supported mammary tumor growth in mice [5].

The significant role of the microbiome in oncogenesis and treatment is emphasized by the fact that in 2022, polymorphic microbiomes, including gut, oral, skin, tumor, or vaginal microbiomes, were integrated into the updated comprehensive concept of “The Hallmarks of Cancer”, summarizing the key characteristics of tumors [6].

The human gut microbiome represents the complex microbiota residing in the human gastrointestinal tract together with microbial genes and metabolites. In addition to important functions in metabolism, nutrient digestion, vitamin synthesis, and protection against pathogens, the favorable composition of the gut microbiota has a crucial impact on maintaining homeostasis in the intestinal microenvironment and shaping the host’s immune system [7]. Techniques used to study the gut and tumor microbiome must be sensitive and specific enough for adequate microbiome characterization. Current methods include mainly genomic-, microscopic- and microbial cultivation-based approaches [8]. A comprehensive analysis of the tumor microbiome in a set of over 1500 samples across seven malignancies, including malignant tumors of the breast, lungs, ovaries, pancreas, bones, brain, melanomas, and adjacent healthy tissues, revealed specific microbial compositions for each tumor type, indicating a correlation with tumor development [9]. Genomic methods such as 16S rRNA sequencing and microscopic imaging using fluorescence in situ hybridization (FISH) were employed to determine the presence of bacteria and bacterial DNA in the tumor microenvironment. Ex vivo bacterial cultivation with fluorescence-labeled d-alanine was used to confirm bacterial viability. Subsequent analysis also revealed correlations between microbial metabolic pathways and clinical parameters [9].

In this Review, we provide current knowledge about the crucial role of the microbiome in tumor development, treatment efficacy with an emphasis on chemo- and immunotherapy, and treatment-induced late effects, including cognitive impairment and cardiotoxicity. Studies focused on identifying potential bacterial biomarkers in predicting therapeutic benefits in cancer patients are gaining significant attention from the wide scientific and clinical community. Microbiota modulation using probiotics, prebiotics, and fecal microbiota transplantation (FMT) may represent a novel approach in cancer patient care, contributing to improved clinical outcomes. A deep understanding of the functional roles of the gut and tumor microbiome and cross-interactions with the human host will allow the application of knowledge about microbial biomarkers in clinical practice.

## 2. Dominant Bacteria-Driven Mechanisms Associated with Cancer Development

The unmodifiable intrinsic and the modifiable or partially modifiable extrinsic factors affect cancer risk [10,11]. Studies showed that beyond microorganisms, numerous risk factors and their complex interplays, such as genetics and inherited mutations, geographical location, gender ratio, age, environmental exposure, and endogenous hormones, contribute to carcinogenesis within human populations [12,13]. A healthy lifestyle, diet, and nutrition play a key role in cancer prevention, and a higher-quality diet might reduce cancer risk [14]. On the other hand, unhealthy lifestyle and obesity are associated with cancer development [15]. Different dietary patterns significantly influence the composition of the gut microbiome [16]. Preparing a diet via the frying process might produce harmful carcinogenic acrylamide as a part of the Maillard reaction, negatively affecting gut microbiome homeostasis [17]. According to the findings, not all HFDs correlated with obesity had a protumorigenic effect. Fish oil HFD did not promote faster mammary tumor growth in a murine model [5].

A comprehensive meta-analysis showed a lower incidence of cancer in vegetarians and vegans [18]. Similarly, Papadimitriou et al. performed an umbrella review of meta-analyses of observational studies to reveal associations between diet or nutrient intake and the risk of 11 primary cancers. Authors observed that dietary products, milk, and calcium were inversely associated with colorectal cancer (CRC), while drinking alcohol correlated positively with breast, colorectal, esophageal, liver, head, and neck malignancies [19].

Geographical provenance affects the composition of bacterial communities residing in the gastrointestinal tract. The gut microbiome of people living in Europe and America showed to be mainly composed of *Dorea*, *Blautia*, *Roseburia*, *Faecalibacterium*, *Ruminococcus*, *Oscillospira*, *Clostridium perfringens*, *Clostridium difficile*, *Staphylococcus aureus*, *Bifidobacterium adolescentis*, *Bifidobacterium catenulatum*, *Akkermansia muciniphila*, and *Bacteroides*. In comparison, intermediate gut diversity and abundance of *Bacteroides* and *Prevotella* were observed in the Asian population [20]. Sun et al. conducted one of the largest studies focused on the impact of 72 factors on gut microbiome variations in Chinese participants from 15 geographic China locations, including 12 provinces and 3 megacities. The authors observed variations in the gut microbiome within different regions of China [21]. The integrated catalog of human gut microbial genes [22] was conducted based on the data obtained from the Human Microbiome Project (HMP) [23], a diabetes study from China [24], and the Metagenomics of the Human Intestinal Tract (MetaHit) project [25]. Country-specific gut microbial differences were revealed between Chinese vs. Danish individuals [22].

Recently, an increasing number of studies confirmed that certain pathogenic microbes contribute to cancer development and progression via impact on DNA in host somatic cells, interrupted cell cycle, increased cell proliferation, and damaged processes responsible for apoptosis [26]. Almost 20% of all cancers might be related to microbial infection [27]. Specific bacteria produce toxins, leading to chronic inflammation with altered cellular processes [28]. However, not all infections with pathogenic microbes lead to cell malignant behavior and cancer development. Genetic heterogeneity of microorganisms and host genetics significantly affect cancer prevalence [29]. Unfavorable microbiota-derived metabolites could exhibit procarcinogenic properties and cause DNA double-strand breaks (DSBs) [30]. Conversely, beneficial bacterial metabolites might exert anti-cancer effects. Microbiota-derived short-chain fatty acids (SCFA) affect not only gut signaling pathways but also organs and tissues via blood circulation [31]. The main SCFA produced in the colon are acetate, propionate, and butyrate [32]. Studies confirmed that butyrate maintains intestinal integrity and reduces chemotherapy-induced mucositis. *Faecalibacterium prausnitzii* is the main producer of butyrate in the colonic microbiota via fermentation of dietary fibers and starch [33,34]. However, a study from 2020 revealed the controversial role of butyrate in patients. Lower baseline concentrations of serum butyrate and propionate correlated with longer progression-free survival (PFS) in French and Italian melanoma patients, respectively. The authors did not observe any significant associations between acetate and PFS [35].

Another bacterial metabolite, reuterine, produced by the probiotic *Lactobacillus reuteri* reduced proliferation and increased CRC cell apoptosis. Reuterin supported protein oxidation, which inhibited ribosomal biogenesis and protein translation [36]. Secondary bile acids, produced by gut microbes mainly by members belonging to the *Clostridium* genus, such as *Clostridium scindens*, *Clostridium hiranonis*, *Clostridium hylemonae*, and *Clostridium sordellii*, have carcinogenic properties [37]. Higher secondary bile acid exposure led to colon carcinogenesis via oxidative stress, mutation, DNA damage, and resistance to cell death [38].

The involvement of microbes and corresponding mechanisms of action have been studied predominantly in gastrointestinal malignancies due to their associations with the gut microbiome. However, mounting studies try to assess the positive/negative impact of bacteria, lipopolysaccharide (LPS), microbiota-derived metabolites, and toxins on cancer development and progression in different cancer types.

### 2.1. Helicobacter pylori

*Helicobacter pylori*, first discovered by Barry Marshall and Robin Warren in 1984 [39], is a gram-negative micro-aerophilic bacterium that might be found in the upper intestinal tract within 50% of the population worldwide [40]. Possible routes of bacterial transmission are via saliva or feces [41]. The colonization of the gastric mucus layer by *Helicobacter pylori* is mediated via adhesins that bind Lewis determinants and mucin 5 (MUC5AC) [42,43]. Bacterial strains can be either cytotoxin-associated gene A (*cagA*) positive or *cagA* negative due to inserted cag pathogenicity island (cagPAI) containing approximately 32 genes [44]. Infection with *Helicobacter pylori* promotes DNA DSBs and induces host genomic instability. The accumulation of DSBs was higher in the case of cagPAI-positive bacterial strains, and the presence of a cagPAI can double the risk of gastric cancer incidence [45]. Within cagPAI, microsyringe (needle-like pilus) coded genes formed the Type IV secretion system (T4SS), which plays a role in CagA oncoprotein translocation into gastric epithelial cells [46]. Oncoprotein is responsible for disrupted epithelial tight junctions and lost apical-basolateral polarity in cells via CagA interaction with partitioning-defective 1 (PAR1)/microtubule affinity-regulating kinase (MARK). CagA prevents PAR1 phosphorylation mediated by atypical protein kinase C (aPKC), resulting in PAR1 dissociation from the membrane [47]. In the case of gastric cancer, only *Helicobacter pylori* strains with CagA virulence factor are associated with cancer development [29]. In addition, this microorganism produces urease, which neutralizes gastric acid in the stomach via produced ammonia from urea. Urease activity contributes to the tolerability of bacterium in the acidic environment of the stomach [48]. Another unique protein secreted by *Helicobacter pylori* is vacuolating cytotoxin A (VacA). This toxin is called “multi-functional” and affects mitochondria, cell junctions, and endocytic compartments. VacA is capable of insertion into the host cell membrane (via the p33 domain) with internalization and formation of anion-selective channels (pores) in membranes with cell destruction [49,50]. Neutrophil-activating protein (NapA) produced by *Helicobacter pylori* participates in pathogen protection and promotes gastric inflammation. NapA activity induces innate and adaptive immune responses and activates immune cells, including neutrophils, monocytes, and mast cells, leading to IL-12 and IL-23 production [51,52].

### 2.2. Fusobacterium nucleatum

*Fusobacterium nucleatum* is an anaerobic bacterium residing in the human gut and oral microbiome, where it co-exists with other microorganisms [53]. In 2012, two individual studies found enrichment of *Fusobacterium nucleatum* in CRC compared to adjacent tissue samples via RNA and whole-genome sequencing [54,55]. This bacterium is linked not only to CRC but also to other human diseases such as periodontal diseases, dental pulp infections, halitosis, oral cancer, infections of the respiratory tract, appendicitis, cardiovascular disease, pregnancy disorders, breast cancer, and rheumatoid arthritis [56,57,58]. FadA and Fap2 represent the key virulence factors of *Fusobacterium nucleatum*. The role of FadA is mediating the attachment and binding to host cells [59]. FadA binds to the specific binding site of E-cadherin and causes bacterial invasion into host epithelial cells [60]. Specific mechanisms of how *Fusobacterium nucleatum* supports inflammation and CRC tumorigenesis result from FadA-induced activation of the Wnt/β-catenin signaling pathway [60,61]. Meta-analysis performed by Wirbel et al. showed the enrichment of the FadA in patient fecal metagenomes detected using sequencing [62]. Later, Li et al. noted that *Fusobacterium nucleatum* supported CRC progression via induced cyclin-dependent kinase 5 (Cdk5), which was involved in the Wnt/β-catenin signaling pathway activation [63]. Virulence factor Fap2 mediates binding to tumor-expressed Gal-GalNAc. Abed et al. demonstrated that Fap2 is attached to host factor Gal-GalNAc overexpressed on CRC cells [64]. Moreover, Parhi et al. hypothesized that *Fusobacterium nucleatum* might bind to Gal-GalNAc-displayed on distant tumors. The study provided several experiments to elucidate the involvement of *Fusobacterium* in breast cancer progression. The results showed that the level of Gal-GalNAc was 4.7-fold higher in breast tumor samples than in adjacent non-tumor tissue. Similarly, *Fusobacterium nucleatum* was detected in breast tumors with higher levels of Gal-GalNAc. In vivo experiments confirmed that *Fusobacterium* ATCC 23726 injection into the tail vein promoted breast tumor growth and the development of lung metastases [65].

### 2.3. Escherichia coli

*Escherichia coli* represents another potential pathogen implicated in CRC. The studies showed a higher prevalence of enteropathogenic *Escherichia coli* (EPEC) in CRC patients compared to healthy controls. Moreover, *Escherichia coli* in patients serotypically and genotypically differed from those in the general population [66]. Pathogenic strains produce toxins, including colibactin, cytolethal distending toxin (CDT), cycle inhibiting factor, and cytotoxic necrotizing factor [67]. CDT is a genotoxin composed of CdtA, CdtB, and CdtC subunits [68], while the CdtB catalytic subunit might support carcinogenesis and host cell transformation in murine experiments [69]. The results from clinical studies showed that colibactin coded by Pks island was observed mainly in CRC patients [67]. Veziant et al. reviewed that colibactin-associated *Escherichia coli* were predominantly present in the colonic mucosa of CRC patients, where they promoted tumorigenic processes [70]. According to the findings, *Escherichia coli* strains and their metabolites promote DSBs, chromosome abnormalities, and cell cycle arrest in host cells [71,72,73]. On the other hand, *Escherichia coli* Nissle 1917 strain, also harboring Pks island, did not promote DSBs in host epithelial cells [74].

### 2.4. Salmonella

Microbial products of typhoid toxin and toxins like nitroso-chemical compounds produced by *Salmonella typhi* might be responsible for the potential development of tumors on the side of infection. The infection with *Salmonella Paratyphi A* caused DNA damage in gallbladder organoids. The experimental results supported associations between *Salmonella*, epithelial cell invasion, initiating malignant transformation, and gallbladder carcinogenesis [75]. Moreover, severe bacterial infection with *Salmonella* might contribute to CRC development [76,77]. *Salmonella* secreted AvrA, a multifunctional protein that activates Wnt and STAT3 signaling pathways, resulting in enhanced proliferation of CRC cells [78]. In vivo experiments showed that AvrA regulates several other pathways, including mTOR, NFκB, oxidative phosphorylation, platelet-derived growth factors, vascular endothelial growth factor, and mitogen-activated protein kinase signaling pathway [79]. AvrA inhibits macrophage death, leading to innate immune signaling blockade. Therefore, AvrA might establish a stable niche for intracellular *Salmonella* where the pathogen avoids adaptive immune responses [80]. Furthermore, animal studies confirmed that non-typhoidal *Salmonella* preferentially infected transformed colon cells and increased the risk of colon carcinogenesis [81]. Effector AvrA protein is crucial for inflammation, anti-apoptosis, and cell proliferation [79].

### 2.5. Bacteroides fragilis

The enteric pathogen known as enterotoxigenic *Bacteroides fragilis* (ETBF) secretes toxin (BFT) coded by the *bft* gene [82]. ETBF strains are implicated mainly in acute diarrheal diseases, but the studies highlight the microbe’s participation in CRC [83]. In vitro experiments documented that BFT increased the production of ROS, induced DNA damage, and promoted tumorigenesis [83]. This toxin is responsible for the damaged epithelial barrier and activated STAT3/Th17 immune responses [84,85]. Geis et al. demonstrated that colonization with ETBF-induced Th17 polarization contributes to carcinogenesis in the murine model [86]. Similarly, BFT supported carcinogenic cascade via activated NFκB in epithelial cells within the distal colon, leading to myeloid cell-dependent distal colon carcinogenesis [87]. A higher level of the *bft* gene was documented in colonic mucosa from late-stage (III/IV) CRC patients than in early stage (I/II) CRC patients [88].

### 2.6. Staphylococcus aureus

This gram-positive bacterium produces several toxins and virulence factors, including Staphylococcal enterotoxin A (SEA) and Staphylococcal enterotoxin B (SEB) [89]. In most cases, *Staphylococcus aureus* is responsible for developed bacteremias in patients with hematologic malignancies [90,91]. Acute myeloid leukemia (AML) cell line revealed increased proliferation after treatment with SEA and SEB virulence factors in vitro [92]. In contrast, an experimental study on glioblastoma cells showed that SEB reduced smad2/3 expression and decreased cancer cell proliferation [93]. Similarly, SEB reduced cancer cell proliferation in U266 cells [94]. Nevertheless, *Staphylococcus aureus* eradication in patients with cutaneous T-cell lymphoma resulted in clinical benefit. Data showed that Staphylococcal enterotoxins did not directly promote T-cells but supported the interactions between malignant and benign T cells, resulting in high levels of IL-10 expressed by malignant T cells [95].

### 2.7. Campylobacter jejuni

The presence of *Campylobacter* is associated with inflammation and implicated in the activation of mTOR signaling and neutrophil infiltration [96]. *Campylobacter* colonizes the intestinal tract due to adherence of *Campylobacter jejuni* CadF and FlpA adhesins to fibronectin. Both adhesins are responsible for physical contact with host cells, contributing to bacterial adherence, invasion, and cell signaling [97]. CDT produced by *Campylobacter* induces DNA damage via DSBs. He et al. demonstrated that human isolates of *Campylobacter* 81–176 supported CRC tumorigenesis, the development of larger tumors, and an altered gene expression profile in the murine model [98].

### 2.8. Desulfovibrio

*Desulfovibrio*, belonging to sulfate-reducing bacteria (SRB), might participate in CRC development via hydrogen sulfide (H_2_S) production. Higher concentration of H_2_S damages DNA, leading to genomic and chromosomal instability [99,100]. Kapral et al. found that LPS from *Desulfovibro desulfuricans* altered the activity of *p65* and *IκBα* genes in Caco-2 colon cancer cells [101]. Moreover, *Desulfovibrio* abundance was significantly higher in patients with advanced gastric cancer (stage IV). Liu et al. aimed to investigate the mechanism of how *Desulfovibrio* promotes gastric cancer and conducted an in vitro experiment with HT-29 cells treated with H_2_S. The results showed that H_2_S promoted NO, IL-1β, and IL-18 production implicated in inflammation [102].

### 2.9. Porphyromonas

Recently, the results from several studies proposed that *Porphyromonas gingivalis*, as an oral pathogen, is implicated in pancreatic and oral tumorigenesis [103,104]. *Porphyromonas* LPS increased gingival stem/progenitor cell proliferation [105]. Gingipains (proteases) secreted by *Porphyromonas gingivalis* are involved in oral cell Notch-1 activation and PLA2-IIA production [106]. Olsen et al. reviewed the potential relationship between *Porphyromonas gingivalis* and oral squamous cell carcinoma. This pathogen increased the levels of specific receptors on carcinoma cells and gingival keratinocytes. *Porphyromonas* also promoted epithelial-to-mesenchymal transition (EMT) via phosphorylation of HSP27 and activated metalloproteinase-9 and IL-8 in carcinoma cells in vitro [104].

## 3. Microbiome and Treatment Efficacy

**Figure 1:**
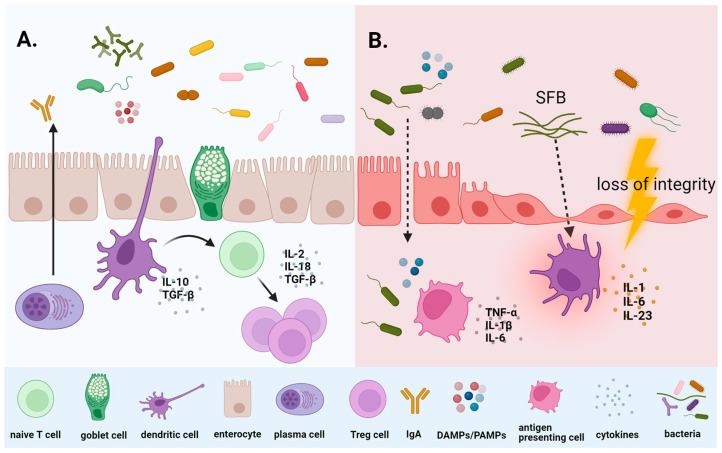
Microbial homeostasis (**A**) and intestinal dysbiosis induced by cancer and corresponding treatment (**B**). The intact commensal microbiota helps to maintain the balance between pro- and anti-inflammatory responses of the immune system (**A**). Chemo- and radiotherapy-associated dysbiosis and mucosal barrier disruption might lead to bacterial translocation, pro-inflammatory cytokine release, and mucosal inflammation (**B**). Abbreviations: DAMPs, damage-associated molecular patterns; IgA, immunoglobulin A; PAMPs, pathogen-associated molecular patterns; SFB, segmented filamentous bacteria; TGF-β, transforming growth factor-beta; TNF-α, tumor necrosis factor-alpha; Treg, regulatory T cells.

In cancer patients, several factors influence the gut microbiome composition. In addition to the malignant disease and the impact of genetic-, diet- and lifestyle-related factors, the administration of antibiotics, immunosuppressants, supportive agents, and especially anti-cancer treatment play a role. Chemotherapy, similar to radiotherapy, heavily disrupts the balance in the microbial environment and leads to gut dysbiosis (Figure 1).

Recently, the association between the gut microbiome, the efficacy, and the toxicity of anti-cancer treatment became the perspective trend in cancer research. Growing evidence suggests that a patient’s microbiome can activate the patient’s immune system and influence the response to various treatment modalities, especially chemotherapy and immunotherapy. Pilot results from animal studies in 2013 and 2015 revealed a weak response to cisplatin in germ-free or antibiotic-treated animals [107] and the importance of commensal *Bifidobacteria* in the anti-tumor immune response and the function of anti-programmed death-ligand 1 (PD-L1) and anti- cytotoxic T lymphocyte-associated antigen-4 (CTLA-4) antibodies [108,109]. These observations were later supported in patient cohorts, showing differences in the microbiome of patients responding and not responding to immunotherapy.

### 3.1. Microbiome and Chemotherapy

Chemotherapy still represents the cornerstone in the comprehensive treatment of cancer, playing a pivotal role in impeding the growth and spread of malignant cells throughout the body. This therapeutic approach either aims to eradicate cancer cells entirely or alleviate symptoms and enhance the quality of life of cancer patients. Indications for chemotherapy vary widely, encompassing a spectrum of cancers at different stages [110]. Chemotherapy may be employed as a neoadjuvant treatment to shrink tumors before surgery or as an adjuvant therapy to eliminate residual cancer cells post-surgery or radiation and/or for the treatment of metastatic disease [111]. Chemotherapeutic agents interfere with the various stages of the cell cycle, impeding DNA synthesis, replication, and cell division. While this process primarily targets rapidly dividing cancer cells, it can also affect normal, healthy cells in the body, leading to a range of side effects [112].

Effectivity varies across different cancer types and individual cases. Some cancers, including malignant lymphomas and/or germ cell tumors, respond remarkably well to chemotherapy, leading to cure, remission, or at least a significant reduction in tumor size, while others may exhibit resistance [113,114]. The personalized nature of chemotherapy regimens, tailored to specific cancer types and patient profiles, underscores the ongoing pursuit of optimizing treatment outcomes in the challenging landscape of cancer care [115].

Toxicity is a significant consideration in chemotherapy, and the treatment’s success must be carefully balanced against its potential adverse effects. Common side effects include fatigue, nausea, hair loss, and decreased blood cell counts, which can result in susceptibility to infections and anemia. Management of these side effects is integral to ensuring the well-being of patients receiving chemotherapy [115].

Groundbreaking findings from studies on animal models have highlighted the beneficial role of commensal bacterial species in modulating the efficacy of chemotherapeutic agents. Iida et al. demonstrated a link between gut microbiota and ROS release, resulting in impaired survival and reduced efficacy of oxaliplatin chemotherapy in germ-free or antibiotic-treated animals [107]. The restoration of chemotherapy efficacy occurred after the administration of bacterial LPS [107]. In 2017, the TIMER mechanism (translocation, immunomodulation, metabolism, enzymatic degradation, and reduced diversity with ecological variation) was proposed, describing the effects of the gut microbiota on chemotherapy [116].

Li et al. described the differences between bacteria residing in the gut microbiome of healthy people and patients with CRC, esophageal, and gastric cancer receiving chemotherapy. Bacterial taxa *Butyricicoccus pullicaecorum*, *Faecalibacterium prausnitzii*, *Roseburia faecis*, *Clostridium clostridioforme*, *Blautia producta*, and *Bifidobacterium adolescent* were prevalent in control stool samples. On the other hand, the enrichment of *Akkermansia muciniphila*, *Bacteroides fragilis*, *Escherichia coli*, *Clostridium hathewayi*, and *Alistipes finegoldii* was seen in the patient microbiome. The results showed that a higher *Roseburia faecis* correlated with a better therapy response [117]. Another study noted that *Fusobacterium nucleatum* plays a role in oxaliplatin chemoresistance among CRC patients by activating the innate immune system [118]. Bacterial taxa, including *Faecalibacterium*, *Clostridiales*, and *Phascolarctobacterium*, were decreased in the gut microbiome of advanced CRC patients treated with the FOLFIRI regimen. Significantly elevated amounts of *Veillonella*, while the reduction in *Prevotella*, *Faecalibacterium*, and *Clostridiales* were documented in postoperative gut microbiome from patients treated with the XELOX regimen [119]. A recent analysis by Tintelnot et al. analyzed the level of tryptophan metabolite indole-3-acetic acid (3-IAA) and 3-IAA gut producers in patients with pancreatic ductal adenocarcinoma who responded or did not respond to FOLFIRINOX. From fifteen 3-IAA producers, *Bacteroides fragilis* and *Bacteroides thetaiotaomicron*, as producers of 3-IAA, were enriched in chemotherapy responders [120].

The important role of the microbiome was also confirmed in cyclophosphamide treatment. Administration of cyclophosphamide significantly altered the composition of the small intestine microbiota, resulting in a decrease in the abundance of bacterial species from the Firmicutes phylum in experimental tumor-bearing mice [121]. The microbial barrier of the small intestine became more permeable to gram-positive bacteria (*Lactobacillus johnsonii*, *Lactobacillus murinus*, and *Enterococcus hirae*), leading to their translocation from the intestine to lymphatic organs. The results confirmed that the gut microbiota shapes the anti-tumor response induced by cyclophosphamide via stimulation of a specific subset of pathogenic Th17 cells (pTh17) and memory Th1 immune responses [121]. Dizman et al. revealed the association between *Barnesiella intestinihominis* and clinical benefits from targeted therapy in metastatic renal cell carcinoma [122]. Moreover, the anti-tumor immunomodulatory effect of this bacterium on chemotherapy was found in the colon [123].

Bacteria within tumors were capable of inactivating the drug gemcitabine into its inactive form in mouse-bearing CRC. Drug metabolism is associated with the expression of the isoform of the bacterial enzyme cytidine deaminase, primarily observed in *Gammaproteobacteria*. According to the results, antibiotic therapy with ciprofloxacin led to overcoming drug resistance in experimental animals. Up to 76% of patient samples with pancreatic adenocarcinoma tested positive for *Gammaproteobacteria*, suggesting that intratumoral bacteria may influence sensitivity to gemcitabine treatment [124]. Moreover, significantly higher intratumoral *Fusobacterium nucleatum* predicted poor chemotherapeutic response in patients with esophageal squamous cell carcinoma [125].

Sims et al. analyzed changes in gut microbiome in response to chemoradiation in patients with cervical cancer. *Porphyromonas*, *Porphyromonadaceae*, and *Dialister* were overrepresented in fecal samples from short-term survivors, whereas *Escherichia/Shigella*, *Enterobacteriaceae*, and *Enterobacteriales* dominated in long-term survivors [126].

Metagenomic analysis focused on the association of the gut microbiome with the response to neoadjuvant chemoradiotherapy revealed differential relative abundances of several bacterial taxa before and after neoadjuvant chemotherapy in rectal cancer patients. Similarly, differences in microbiota composition were identified between patients responding to treatment and non-responders. The responders exhibited a higher prevalence of *Shuttleworthia*, whereas non-responding patients showed an abundance of *Clostridiales* [127]. Multi-omics analysis revealed that *Bacteroides vulgatus*-associated nucleotide biosynthesis decreased response to preoperative neoadjuvant chemoradiotherapy in patients with locally advanced rectal cancer via upregulated genes involved in DNA repair processes [128]. A recent analysis of patients with muscle-invasive bladder cancer treated with neoadjuvant chemotherapy revealed the presence of favorable microbes in patients with a complete response. However, data did not confirm any specific microbial biomarkers of therapy response [129]. Yi et al. found associations between the gut microbiome of locally advanced rectal cancer patients and different responses to neoadjuvant chemoradiotherapy. Significant enrichment of *Roseburia*, *Dorea*, and *Anaerostipes* was documented in responders, while *Coriobacteriaceae* and *Fusobacterium* were overrepresented in non-responding patients [130].

### 3.2. Microbiome and Immunotherapy

Immunotherapy represents a major advance in the clinical management of several cancers. Over the last decade, it has revolutionized the treatment of solid and hematologic malignancies, even those associated with a poor prognosis. The most widely used are immune checkpoint inhibitors (ICI), developed to enhance the activity of the body’s own immune cells against cancer cells [131]. ICI includes monoclonal antibodies designed to block the immune regulators, CTLA-4 (ipilimumab, tremelimumab), programmed death-1 (PD-1) (nivolumab, pembrolizumab), and PD-L1 (atezolizumab, avelumab, durvalumab) expressed in the cancer cells, with the consequent cytotoxic immune response. While these immunotherapies have improved patient outcomes in many clinical settings, they can induce toxicity, specifically immune-related adverse events. Commonly experienced adverse effects include cutaneous, musculoskeletal, intestinal, endocrine, and pulmonary, while cardiovascular, hematologic, renal, and neurological occur much less frequently [132]. However, the cardiovascular toxicity of ICI is of particular concern, given their impact on the morbidity and mortality of cancer patients [133]. Myocarditis is a severe complication of ICI with a high fatality rate that most frequently develops during the first 12 weeks of treatment, although late cases may occur [134,135].

Currently, an increasing number of studies are addressing the impact of the microbiome on the efficacy of immunotherapy, and accumulating evidence confirms that modulating the gut microbiome in favor of a favorable composition proves to be a promising trend in improving the response to immunotherapy using ICI [136]. The introduction of immunotherapy represents a key step in cancer treatment, with blocking CTLA-4, PD-1, and PD-L1 checkpoint pathways helping to restore the anti-tumor immune response [137]. The fundamental mechanisms underlying the relationship between the microbiome and the response to immunotherapy include increased infiltration of tumor immune cells, maturation of dendritic cells, and the production of IL-12, which promotes increased differentiation of Th cells and immune activation in the tumor microenvironment. It also involves the expansion of cytotoxic CD8 cells associated with the upregulation of perforin and serine protease granzyme, leading to apoptotic destruction of tumor cells [138,139].

Vetizou et al. highlighted the relationship between *Bacteroides thetaiotaomicron-* and *Bacteroides fragilis*-specific T-cell responses and the efficacy of CTLA-4 blockade in a mouse model and in cancer patients. Oral administration of *Bacteroides fragilis* or immunization with corresponding polysaccharides restored the response to immunotherapy in antibiotic-treated mice, previously non-responding to treatment [108]. *Akkermansia muciniphila* and *Prevotella* improved PD-1 blockade efficacy in mouse-bearing CRC, whereas *Bacteroides* correlated with poor response [140]. Specific bacterial members, including *Lachnoclostridium*, *Parabacteroides*, *Lachnospiraceae*, *Ruminococcaceae*, *Dialister*, and *Flavonifractor* were enriched in CRC patients responding to PD-1/PD-L1 blockade compared to taxa *Coprococcus*, *Bacteroides*, *Parabacteroides*, and *Subdoligranulum* which dominated in non-responders. In the case of gastric cancer patients, the prevalence of *Prevotella*, *Bifidobacterium*, and *Lachnospiraceae* was observed in immunotherapy responders, while *Megamonas*, *Butyricimonas*, *Lachnospiraceae* UCG 001, and *Agathobacter* in non-responders [141].

In HER2-positive breast cancer patients, a decrease in *Lachnospiraceae*, *Prevotellaceae*, *Turicibacteraceae*, and *Bifidobacteriaceae*, together with lower alpha diversity were observed in non-responders to trastuzumab. Feces transferred from responding and non-responding patients led to recapitulated trastuzumab response in mice-bearing breast tumors [142]. Higher alpha diversity in the gut microbiome of triple-negative breast cancer patients before chemotherapy positively correlated with patients' complete response [143].

Lee et al. revealed that specific *Bifidobacterium bifidum* strains acted synergically with PD-1 blockage or oxaliplatin treatment in animal models with lung cancer. According to the results, immunostimulation and INF-γ production within the tumor microenvironment might be associated with improved response to immunotherapy after *Bifidobacterium* supplementation [144]. The analysis of patients with non-small cell lung carcinoma or renal carcinoma indicated a correlation between the relative abundance of *Akkermansia muciniphila* and the clinical response to immunotherapy. *Akkermansia* was overrepresented in stool samples from responding patients to treatment, and oral supplementation with *Akkermansia muciniphila* alone or combined with *Enterococcus hirae* restored the efficacy of PD-1 blockade in germ-free and antibiotic-treated mice after FMT from refractory patients [145]. Vernocchi et al. confirmed that an increased amount of *Granulicatella* positively correlated with nivolumab response in patients with non-small cell lung carcinoma [146]. Another study showed significantly reduced alpha diversity and absence of *Ruminococcaceae* UCG 13 and *Agathobacter* in patients with advanced non-small cell lung carcinoma who received antibiotics before ICI. However, higher levels of both microbes positively correlated with objective response rate (ORR) and PFS > 6 months in ICI-treated patients who were not supplemented with antibiotics [147].

Metagenomic analysis of a cohort of 112 melanoma patients revealed higher bacterial diversity enriched with *Faecalibacterium* species in stool samples from patients responding to PD-1 blockade. On the contrary, stool samples from non-responding patients were enriched with *Anaerotruncus colihominis* and *Escherichia coli*. The presence of *Faecalibacterium* was positively associated with longer PFS after PD-1 blockade, while patients with higher levels of *Bacteroidales* showed shorter PFS [148]. A cohort of metastatic melanoma patients showed microbiome alterations according to PD-1 blockade response. Stool samples from responding patients were enriched in *Bifidobacterium adolescentis*, *Bifidobacterium longum*, *Collinsella aerofaciens*, *Enterococcus faecium*, *Klebsiella pneumoniae*, *Parabacteroides merdae*, and *Veillonella parvula*, while *Roseburia intestinalis* and *Ruminococcus obeum* dominated in non-responders [149].

Botticelli et al. studied whether gut-related metabolome was implicated in immunotherapy response. The gut metabolome of long responders was characterized mainly by SCFA, nicotinic acid, and lysine. On the other hand, alkanes, ketones, aldehydes, and p-cresol were prevalent in the metabolome of early progressors [150].

## 4. Microbiome and Therapy-Induced Late Effects

Cancer treatment, especially chemotherapy, causes a range of late complications in survivors, including neurological, ophthalmological, pneumatological, cardiological, and nephrological complications or issues linked to infertility and necrosis of the femoral head [151]. Cancer survivors experience disruption of the immune system correlated with therapy or the malignant disease. Considering that gut microbiome composition is crucial for shaping the immune system, the associations between the gut microbiome and treatment-induced late effects are gaining attention. In this context, a lot of evidence has documented the impact of dysbiosis on the function of the nervous and cardiovascular systems [152], and numerous clinical trials are still ongoing (Table 1).

### 4.1. Microbiome and Treatment-Induced Cognitive Impairment

The brain is highly sensitive to microbial disharmony, and the altered composition of the intestinal microbiome significantly affects the physiology and functions of the nervous system. Changes in the microbiota-host relationship affect the enteric nervous system and activate neuroimmune signaling pathways, influencing brain development and functioning [153]. An in vivo study showed a constitutive activity of the host microbiome on the innate brain immune system, demonstrating that microbial metabolites regulated microglial homeostasis in experimental animals [154]. Ciernikova et al. proposed mechanisms linking chemotherapy-induced changes in the intestinal microecosystem and cognitive impairment in cancer patients [155].

Microbial signals, including structural bacterial components or microbiota-derived metabolites, can influence distant organs directly or through neural and hormonal signaling. Systemic inflammation induced by intestinal dysbiosis can increase the stress-activated “hypothalamus-pituitary-adrenal” (HPA) axis [156]. Mechanistic studies have revealed pathways through which communication occurs along the microbiome-gut-brain axis. The gut microbiota produces microbiota-derived metabolites such as SCFA, trimethylamine *N*-oxide (TMAO), endotoxins, and amino acids, circulating in the blood to the brain and affecting nervous functions. In addition to the role of SCFA in maintaining the integrity of the intestinal membrane and mucin production, SCFA´s involvement in signaling between the microbiome, gut, and brain via immune, endocrine, and humoral pathways is intensively studied [157]. Certain strains of gut bacteria can secrete neurotransmitters such as acetylcholine, gamma-aminobutyric acid (GABA), tryptophan, and serotonin. GABA is a neurotransmitter that helps to maintain the healthy functioning of the brain and nervous system [158]. Metagenomic and metabolomic analyses showed that not only higher levels of *Fusobacteium nucleatum* but also reduced SCFA and decreased GABA biosynthesis were implicated in late-onset CRC [159]. Serotonin is a neurotransmitter that plays a crucial role in mood and learning, showing major production in the gastrointestinal tract [160,161]. Intestinal dysbiosis and elevated levels of pro-inflammatory microorganisms lead to the activation of innate and adaptive immune cells. Moreover, microbial translocation may result in systemic inflammation [162], and pro-inflammatory cytokines can be transported to the brain through the bloodstream, causing neuroinflammation.

Cancer treatment can lead to cognitive impairment associated with memory deficits, attention problems, information processing, and decision-making abilities. These negative impacts can persist long-term after the end of treatment, significantly affecting the lives of survivors. A comparison of cognitive functions in 581 breast cancer patients and 364 healthy individuals in the control group revealed that more than one-third of patients in the chemotherapy group experienced cognitive dysfunction that persisted for at least 6 months post-treatment [163]. The relationship between chemotherapy, radiotherapy, and decreased cognitive functions was also confirmed in a cohort of 155 testicular cancer patients [164].

According to recent findings, chemotherapy-induced cognitive impairment and neuroinflammation may be associated with altered gut microbiome composition after the treatment. One of the first studies pointing to this association was an experimental finding showing chemotherapy-associated changes not only in the gastrointestinal tract but also in the blood and brain of experimental animals [165]. The authors noted that anorexia slowed growth, cognitive function disorders, increased levels of pro-inflammatory processes, damage to the morphology of the intestinal membrane, increased release of endotoxins into the blood, and reduced microbial diversity were observed after paclitaxel administration [165].

Polyphenols, known as plant-derived natural compounds, are promising therapeutic strategies for reducing oxidative stress and neuroinflammation via their anti-inflammatory and anti-oxidative effects [166]. Li et al. reviewed that polyphenols as a prebiotic substrate might keep a healthy gut microbiome via supported beneficial bacteria and promote a neuroprotective effect [167]. Moreover, the biotransformation of dietary polyphenols by favorable gut microbial composition supports cognitive function via produced neurotransmitters and bioactive metabolites [168]. These metabolites reach the brain via the crossed intestinal and blood–brain barrier [169]. Preclinical experiments documented that polyphenols and polyphenol-rich sources decreased pathogenic *Clostridium histolyticum* and *Clostridium perfringens*, whereas increased beneficial bacteria, including *Bifidobacteria* and *Lactobacilli* [170].

Cross et al. documented the neuroprotective effect of diet fiber in female C57Bl/6 mice treated with 5-fluorouracil (5-FU) via alleviated 5-FU-induced neuroimmune changes. A fiber-rich diet altered gut microbial composition increased *Akkermansiaceae*, *Bacteroidaceae*, and elevated propionate production [171]. Pelvic irradiation led to changes in the gut microbiome, intestinal barrier, neuronal maturation, neuronal survival, and developed neuroinflammation in irradiated Sprague Dawley rats. Consequently, gut dysbiosis might result in the entering of pathogenic bacteria into the bloodstream and brain, inducing several changes in both neuronal and glial compartments [172].

In 2022, Smith et al. observed that antibiotic pretreatment 4 weeks before CAR-T cell therapy correlated with worse survival and developed neurotoxicities in patients with hematologic malignancies. A higher fecal abundance of *Akkermansia*, *Ruminococcus*, *Bacteroides*, and *Faecalibacterium* was associated with an improved therapy response [173]. A very recently published analysis of peripheral blood in 142 cured oncology patients with testicular germ cell tumors pointed to the association of the biomarker sCD14, which plays a role in microbial translocation and monocyte activation via LPS, with reduced cognitive functions after cisplatin treatment [174].

### 4.2. Microbiome and Cardiovascular Toxicity

The expanding range of cancer therapeutics has led to a broad spectrum of cardiovascular complications diagnosed in patients during and after cancer therapy. Moreover, high cardiotoxicity is the reason for treatment discontinuation. Cancer therapy-related cardiovascular toxicity includes cardiomyopathy, heart failure, myocarditis, coronary artery disease, peripheral vascular disease, hypertension, arrhythmias, pericardial, valvular heart diseases, and thromboembolism [175,176,177,178]. These complications are linked to chemotherapy (such as anthracycline cytostatics and platinum derivates), targeted agents (monoclonal antibodies and tyrosine kinase inhibitors), immunotherapy (including mainly ICI), and radiation therapy (to the left chest or mediastinum). Significant excesses in mortality risk associated with treatment-related complications, including cardiac causes, exist up to 2 years after the initial cancer diagnosis [179]. Several studies have confirmed that cancer patients have a 2–6-fold higher risk of cardiovascular morbidity and mortality than the general population [180,181].

The relative risk of both arterial and venous thromboembolism is significantly higher in cancer patients compared with the general population [182]. A recent study comprising 12,414 ARIC (Atherosclerosis Risk In Communities) participants monitored for decades showed that cancer patients had a 52% higher risk of heart failure and a 22% higher risk of sudden stroke compared to patients without a cancer diagnosis [183].

Study results indicate that gut dysbiosis may promote the development of atherosclerosis and heart failure [184]. LPS, a component of the cell wall of gram-negative bacteria, promotes the formation of inflammatory cytokines in cardiovascular diseases [185]. A comparison between healthy individuals and patients with heart failure revealed a different composition of the microbiome, reduced beta diversity of the gut microbiota, altered gut barrier permeability, bacterial translocation, increased levels of circulating LPS, and endotoxins in the patients’ blood [186,187].

Chemotherapy-induced intestinal barrier disruption results in the release of bacterial LPS into the bloodstream. As noted, mammalian endotoxin receptor TLR4 is associated with doxorubicin-induced cardiopathy [188]. Wang et al. described the involvement of TLR4 in the damage to the heart, kidneys, liver, and intestines caused by doxorubicin. Research on mouse models has suggested that modulation of the gut microbiota or inhibition of TLR signaling could be an effective approach to mitigate doxorubicin toxicity, and there is consideration for possible implementation for other chemotherapeutics [189]. The involvement of microorganisms in metabolic pathways associated with cardiovascular diseases and the production of bacterial metabolites, including TMAO, SCFA, secondary bile acids, and uremic toxins, implies an active connection between the microbiome and cardiotoxicity induced by cancer therapy.

TMAO arising from intestinal microbiota is a novel biomarker linked to atherosclerosis and risk of major adverse cardiovascular disease events and death in animals and humans [190,191,192,193]. TMAO levels have also been shown to correlate with pro-inflammatory state [194]. Due to its connection to dietary intake, TMAO could be influenced by intermittent fasting, and its change highlights the possibility that fasting may also beneficially alter the microbiome, at least during caloric restriction, if not for a more extended period of time after the completion of fasting. Benefits on metabolic health parameters, lower risk of coronary heart disease and depression, and cognitive performance improvement may be reached by a 24 h water-only fasting intervention in apparently healthy individuals [195]. The mechanisms and results of those changes should be investigated further in longer-term studies with repeated episodes of intermittent fasting. However, several other studies have not confirmed the correlation between gut-microbiota metabolites and atherosclerosis [196,197].

BMS-1 is a molecule inhibitor of PD-1/PD-L1 interaction with a similar effect as immunotherapy via PD-1/PD-L1 blockade, suggesting its application as a replacement for immunotherapy [198]. Chen et al. documented gut dysbiosis with a prevalence of *Escherichia/Shigella*, *Ruminococcaceae*, and depleted *Prevotellaceae* and *Rikenellaceae* with low butyrate production in BMS-1-induced cardiotoxicity. Feces transferred from BMS-1-treated mice induced apoptosis of cardiomyocytes in antibiotics-pretreated recipient mice. Moreover, colonization with *Prevotella loescheii* and butyrate supplementation reduced BMS-1-induced cardiotoxicity. The results proposed that gut dysbiosis with the prevalence of unfavorable bacteria might contribute to cardiotoxicity [199].

Anti-heart failure therapies are preferred in the treatment of doxorubicin-induced cardiotoxicity. However, strategies involved in the prevention of doxorubicin-induced cardiotoxicity are still limited [200]. Therefore, Lin et al. hypothesized that dietary polyphenols within yellow wine might alleviate the cardiotoxic effect in doxorubicin-treated rats via microbiota regulation. Pretreatment with yellow wine polyphenolic compound reversed gut dysbiosis, decreased doxorubicin-induced cardiotoxicity, elevated cardiac and mitochondrial function, and reduced inflammation [201].

## 5. Microbiota Modulation by Probiotics, Prebiotics, and FMT in Cancer Patients

**Figure 2:**
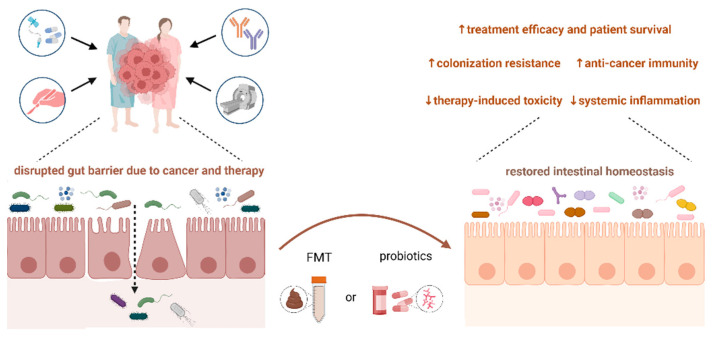
The impact of microbiota modulation using probiotics or FMT in cancer patients. Restoration of favorable microbiome composition and increased integrity of the intestinal barrier might lead to improved cancer patient outcomes via increased efficacy and reduced toxicity of cancer treatment. Abbreviations: FMT, fecal microbiota transplantation.

Mounting evidence highlights the emerging role of gut microbiota modulation in cancer patients via probiotic and prebiotic administration [202]. FMT from a healthy donor or treatment-responding patient quantitatively and qualitatively surpasses the supplementation with probiotics alone. The safety and efficacy of microbiota modulation in immunosuppressed cancer patients are the subject of intense research, and studies confirm the positive effect of modulation on patient outcomes (Figure 2).

Most results documenting a positive relationship between probiotics and patient outcomes come from studies on CRC patients. A probiotic mixture of *Lactobacillus acidophilus*, *Enterococcus faecalis*, and *Bifidobacterium longum* decreased diarrhea in patients with colorectal and rectal tumors [203]. A meta-analysis involving 2982 cancer patients suggested that the administration of probiotics may represent a cost-effective and safe tool for reducing the incidence of severe infections and diarrhea in the probiotic-supplemented group [204]. However, rare probiotic-related complications have been documented, including catheter bacteremia, fungemia, and positive serial blood cultures [205]. Wardill et al. performed a meta-analysis with 1091 patients who suffered from different primary malignancies, including gynecological, colorectal, and lung cancers, who received probiotics for the prevention of therapy-induced diarrhea. The results showed that probiotic supplementation, mostly with *Lactobacillus* strains, did not reduce or prevent the occurrence of diarrhea [206]. Similarly, Danis et al. noted that probiotics failed to improve diarrhea in patients treated by chemotherapy with/without radiotherapy [207]. In 2021, Rodriguez-Arrastia documented the positive impact of probiotics in 17 studies (85%), while no effects of probiotics on treatment-induced side effects were reported in 3 studies (15%). Studies enrolled in systematic review and meta-analysis were carried out in Asia, Europe, America, and Oceania [208].

Postoperative administration of combined tablets containing *Bifidobacterium infantis*, *Lactobacillus acidophilus*, *Enterococcus faecalis*, and *Bacillus cereus* restored a favorable microbiome composition in stool samples from 100 CRC patients undergoing chemotherapy. Supplemented patients experienced a significant reduction in gastrointestinal toxicity compared to the placebo group [209]. The administration of the probiotic preparation Colon Dophilus™ (a synbiotic with numerous *Bifidobacterium* spp., *Lactobacillus* spp., *Streptococcus thermophilus*, maltodextrin, magnesium stearate, ascorbic acid, and inulin) led to a reduction in the occurrence and severity of diarrhea, as well as a less frequent presence of enterocolitis in a randomized clinical study involving 46 CRC patients treated with irinotecan. Additionally, no infections caused by probiotic strains were recorded during probiotic supplementation [210]. A more recent multicenter validation study focusing on the efficacy of a probiotic mixture containing *Bifidobacterium* BB-12^®^ and *Lactobacillus rhamnosus* LGG^®^ in the prophylaxis of irinotecan-induced diarrhea in 242 patients with metastatic CRC, did not demonstrate a significant difference in the occurrence of grade III/IV diarrhea or overall diarrhea incidence after probiotic supplementation compared to the placebo group. However, subgroup analysis suggested a potential clinical benefit in patients with colostomy, showing a higher incidence of grade III/IV diarrhea and any diarrhea in the placebo group compared to the probiotic group [211]. Probiotic administration also shows potential in mitigating chemotherapy-induced late effects on cognitive functions. Lee et al. demonstrated that the administration of *Lactobacillus rhamnosus* and *Lactobacillus acidophilus* alleviated symptoms of depression, anxiety, and fatigue in CRC survivors [212]. In addition, gut microbiota modification via probiotics might modulate neuroinflammation. In vivo experiments claimed that probiotic supplementation alleviated radiotherapy-induced intestinal damage and reduced neuronal inflammation [213].

The significance of probiotic supplementation is also evident in cases of malignancies other than gastrointestinal, showing a reduction in the severity of oral mucositis after probiotic supplementation in patients with advanced oropharyngeal carcinoma undergoing chemoradiotherapy [214]. A recent study highlighted the beneficial effects of administering *Bacillus clausii* UBBC 07 in preventing the development of grade IV oral mucositis after radiotherapeutic treatment in patients with head and neck tumors [215]. On the other hand, synbiotic supplementation (*Lactobacillus paracasei*, *Lactobacillus rhamnosus*, *Lactobacillus acidophilus*, and *Bifidobacterium lactis* with fructooligosaccharides) via nasoenteric tube starting after the surgical procedure did not affect gut microbiome function and postoperative complications in patients with head and neck cancer [216]. Additionally, DE Sanctis et al. did not observe differences in the incidence of oropharyngeal mucositis in radiochemotherapy-treated head and neck cancer patients supplemented with *Lactobacillus brevis* CD2 lozenges or control sodium bicarbonate mouthwash [217].

In a group of lung cancer patients receiving probiotic supplementation with *Clostridium butyricum*, a lower incidence of chemotherapy-induced diarrhea, nausea, and vomiting was recorded compared to the placebo group [218].

The use of FMT opens up new possibilities in the treatment of intestinal damage caused by radiotherapy, with the potential to improve clinical outcomes in cancer patients [219]. Moreover, patient-to-mice studies documented an improved response to therapy with ICI in mice receiving a fecal transplant from responding patients compared to animals receiving FMT from non-responders. Gopalakrishnan et al. observed that stool samples from melanoma patients responding to PD-1 blockade were enriched in *Clostridiales/Ruminococcaceae*. However, high levels of *Bacteroidales* were detected in patients who were non-responding to ICI. Transfer of stool from responding patients into the intestinal tract of germ-free mice resulted in slowed tumor growth and increased levels of *Faecalibacterium* compared to FMT recipients from non-responders [148]. Fecal microbiome analysis of patients with non-small cell lung and renal cell carcinoma receiving PD-1 blockade showed an improved therapeutic response in patients with an abundance of *Akkermansia muciniphila*. As shown, orally administered FMT from responding patients led to decreased tumor growth in antibiotic-treated animals [145]. Fecal transfer from pancreatic cancer responders led to the development of smaller tumors in mice inoculated with cancer cells compared to the animals colonized with microbiome from non-responding patients to chemotherapy [120]. Baruch et al. documented partially or completely restored the response to reinduction with nivolumab in three metastatic melanoma patients after FMT from two responders with enrichment of *Lachnospiraceae*, *Ruminococcaceae*, and *Veillonellaceae* [220]. Another study on refractory melanoma patients described a positive impact of FMT from responders with an increase in bacterial strains of Actinobacteria and Firmicutes and low Bacteroidetes on pembrolizumab efficacy [139].

## 6. Critical Analysis of the Clinical Utility of a Microbiome-Based Approach

The relationship between microbiome and cancer is mainly based on studies showing an association between the presence of bacteria or their composition and a specific tumor type. However, studies demonstrating a causal association are rare. Here, we found several methodological difficulties since the microbiome appears to be an influencing factor for tumorigenesis rather than an independent causal factor. Challenges arise from the absence of suitable experimental models for examining shifts in the microbiome and determining the changes in the production of microbiota-derived metabolites during carcinogenesis. Additionally, the diverse composition of the animal microbiome, along with factors such as colonization resistance, further complicate our understanding of this intricate process. Although many studies have shown an association between the microbiome and a specific cancer type, characterized bacterial taxa often vary between studies, even when the same type of cancer is involved.

The development of reliable microbiome-based treatment strategies presents a multifaceted set of challenges. A critical hurdle is ensuring the survival of exogenously administered microbial strains in the complex host environment while regulating the production of therapeutic agents to achieve controlled quantities. Factors such as host immune responses, competition with existing microbial communities, and the ability to regulate the production of therapeutic agents need to be addressed in further investigations. Moreover, targeted delivery to specific tissue sites, accurate assessment of therapeutic compounds, and comprehensive understanding of links between microbial metabolites and cancer progression add an additional layer of complexity. These all make providing the causality extremely challenging. Standardizing assessment tools across studies, translating experimental findings into clinical practice, and addressing the ethical considerations associated with microbiome manipulation are integral. In addition, consideration of patient microbiome variability highlights the need for a nuanced and interdisciplinary approach to overcome these challenges.

Another group of limitations is represented by studies with microbiota modulation using pro-, pre-, or post-biotics and FMT. The heterogeneity in the preparation strategy remains a problem, as well as the issue of evaluating the efficiency of colonization and, in particular, the production of bacterial metabolites after microbiota modulation. This heterogeneity is mostly obvious in the case of FMT, showing significant differences in processing, storage, and the route of application.

A big challenge represents the fact that in therapeutic administration, most work has followed a one-fits-all approach, not taking into account the individual composition of the original microbiome, its colonization resistance, the influence of dietary composition, concomitant medication and host factors that can all influence the composition of the microbiome. Moreover, we lack data from randomized trials on the clinical utility of the approach.

An essential factor to consider when planning future studies is the use of relevant clinical endpoints, not just surrogate markers, to assess efficacy. At the same time, the broadest possible translational research involving the collection and characterization of biological material from different time points should be an integral part of intervention studies in order to understand as precisely as possible the interaction between the approaches to modify the microbiome, the markers of this change, and the clinical endpoint to be affected. Formulating strategies for human microbiota modification should follow the same principles as any other drug development, including pharmacokinetic and pharmacodynamic evaluations.

## 7. Conclusions and Future Directions

The role of the gut microbiome in cancer patients is not yet fully elucidated, but its importance is continually confirmed. Advances in complex molecular biology and genetic approaches, along with the development of sophisticated bioinformatic algorithms, have enabled extensive microbial analyses and brought us closer to understanding the true impact of the microbiome on human health. However, microbiome research is closely associated with several challenges, especially standardizing technological procedures for sample collection, including storage and sample processing. It also involves complex analysis of sequencing data and defining causal relationships between changes in microbiome composition and malignant diseases.

Microorganisms can contribute to the initiation and progression of malignancies at both local and systemic levels by influencing the host immune response and producing metabolites and genotoxins by individual bacterial taxa. Clinical studies have confirmed extensive changes in the gut microbiome after undergoing anti-tumor therapy, with the most data available for patients treated with chemotherapy, radiotherapy, and immunotherapy. Simultaneously, the individual composition of the microbiome can activate the immune system and enhance the patient´s response to the administered treatment.

Restoring the balance in the gastrointestinal tract is possible in several ways, opening the opportunity for modulating the gut microbiota to reduce acute and late treatment toxicity and improve therapeutic response. Given the interaction between the gut and tumor microbiome, modulations may also influence the composition of the tumor microbiome. Therefore, personalized determination of the gut and tumor microbiome may represent a potential diagnostic and prognostic tool, and research in the coming years will reveal the most effective and safest ways to modify the microbiome to improve patient outcomes. Despite many challenges, it is highly likely that microbiome research in oncology will increasingly contribute to the diagnosis of cancer and the stratification of patients for the development of more effective, individualized, tumor-specific therapies in the next decade. The implementation of metatranscriptomic and metabolomic approaches, which complement metagenomic analyses, will also be of the highest interest. Importantly, machine learning algorithms might play a significant role, helping to uncover signaling networks for identifying new targets to predict treatment response.

## Figures and Tables

**Table 1:** Microbiome and cancer treatment-induced long-term side effects. The table summarizes the list of ongoing clinical trials evaluating the relationship between gut microbiome changes and the occurrence of late toxicities in cancer survivors.

ClinicalTrials.gov Identifier:	Study Design	Malignancy	Purpose	Patients (*n*)	Intervention Model Description	Study Status
**NCT05155618**	An interventional randomized study, crossover assignment	Prostate cancer	To evaluate the shifts in the microbiome and correlation with changes in cytokines and adipokines, particularly in the context of late-onset toxicity	300 adults	Participants in the intervention group will meet a dietitian and physiotherapist to obtain personalized diet and exercise recommendations. Patients undergoing radiotherapy will receive a 6-month intervention followed by a 6-month follow-up.	Recruiting
**NCT04775355**	An observational prospective study	Prostate cancer	To analyze gut microbiome during androgen deprivation therapy and radiotherapy and reveal changes in the gut microbial community related to late toxicity	30 adults	Participants will complete questionnaires prior to radiotherapy, mid-way through therapy, and after completion of radiotherapy.	Recruiting
**NCT03294122**	An observational prospective study	Head and neck cancer/prostate cancer	To examine how intestinal/salivary microbiomes affect toxicity	400 adults	Anti-cancer therapies will include radiotherapy with possible adjuvant hormone therapies/concomitant chemotherapies. CTCAE will define a grading system for late toxicities.	Unknown
**NCT05349227**	An interventional randomized, open-label study, crossover assignment	Ovarian cancer/ breast cancer/ lung cancer/ gastric cancer	To monitor changes in depression, cognitive function or impairment, sleep-related impairment and analyze the gut microbiome in fecal samples at baseline of study enrollment and month 6 following enrollment	660 adults	Patients will be divided into wrist-worn devices monitored groups where they will receive either 6 months of digital coaching immediately followed by 6 months of monitoring or 6 months of monitoring followed by 6 months of digital health coaching.	Recruiting
**NCT06098404**	An observational prospective study	Cancer	To study the composition of gut microbiome and correlate fatigue incidence with gut microbiome composition	250 adults	The cancer-related cognitive impairment will be monitored using MoCA consisting of 9 questions (focused on memory, attention, language, abstraction, delayed recall, and orientation), while fatigue and cognition with FACS (20-question assessment).	Not yet recruiting
**NCT06050733**	An observational cross-sectional study	Solid cancer	To observe the associations between cognition and fatigue with gut microbiome composition	16 adults	Fatigue and cognition will be evaluated by FACS (20-question assessment), MFSI-SF (30-question assessment), and MoCA consisting of 9 questions in patients receiving standard therapy with PD-1/PD-L1 blockade.	Recruiting
**NCT04691284**	An interventional prospective, single-center, non-randomized, open-label study, single-group assignment	Hematologic malignancies	To compare microbiome alternations with quality of life, spirituality, and cognitive functions	100 adults	Enrolled patients will receive high-dose chemotherapy and hematopoietic cell transplantation or CAR-T cell therapy.	Recruiting
**NCT06088940**	An interventional double-blinded, placebo-controlled, randomized study, parallel assignment	Cancer	To investigate probiotics vs. placebo effect on gut taxons and correlate bacterial operational taxonomic units with gastrointestinal and psychosocial functions	66 adults	Cancer patient survivors will receive one probiotic (*Lactobacillus* and *Bifidobacterium* strains)/placebo (maltodextrin) capsule daily for 12 weeks. The effect of probiotics on diarrhea/gas/bloating/anxiety/fatigue symptoms and cognitive function will be measured.	Not yet recruiting

Abbreviations: CTCAE, The Common Terminology Criteria for Adverse Events; FACS, The Fatigue and Altered Cognition Scale; MFSI-SF, Multidimensional Fatigue Symptom Inventory Short Form; MoCA, The Montreal Cognitive Assessment; PD-1/PD-L1, programmed death-1/programmed death-ligand 1.

## Data Availability

Not applicable.
